# Epidemiology, Clinical Features, and Outcomes of Coccidioidomycosis, Utah, 2006–2015

**DOI:** 10.3201/eid2709.210751

**Published:** 2021-09

**Authors:** Adrienne Carey, Morgan E. Gorris, Tom Chiller, Brendan Jackson, Wei Beadles, Brandon J. Webb

**Affiliations:** University of Utah School of Medicine, Salt Lake City, Utah, USA (A. Carey);; Los Alamos National Laboratory, Los Alamos, New Mexico, USA (M. Gorris);; Centers for Disease Control and Prevention, Atlanta, Georgia, USA (T. Chiller, B. Jackson);; Utah Department of Health, Salt Lake City (W. Beadles);; Intermountain Healthcare, Salt Lake City (B. Webb);; Stanford University School of Medicine, Palo Alto, California, USA (B. Webb)

**Keywords:** antifungal agents, Coccidioides, Coccidioides immitis, Coccidioides posadasii, coccidioidomycosis, epidemiology, fungi, Intermountain Healthcare system, pulmonary infections, United States, Utah

## Abstract

On the basis of a 1957 geographic *Coccidioides* seropositivity survey, 3 counties in southwestern Utah, USA, were considered coccidioidomycosis-endemic, but there has been a paucity of information on the disease burden in Utah since. We report findings from a recent clinical and epidemiologic study of coccidioidomycosis in Utah. To describe clinical characteristics, we identified all coccidioidomycosis cases in an integrated health system in the state during 2006–2015. For epidemiologic analysis, we used cases reported to the Utah Department of Health during 2009–2015. Mean state incidence was 1.83 cases/100,000 population/year. Washington County, in southwestern Utah, had the highest incidence, 17.2 cases/100,000 population/year. In a generalized linear model with time as a fixed effect, mean annual temperature, population, and new construction were associated with regional variations in incidence. Using these variables in a spatiotemporal model, we estimated the adjusted regional variation by county to predict areas where *Coccidioides* infections might increase.

Coccidioidomycosis, also known as Valley fever, is caused by *Coccidioides immitis* and *C. posadasii*, endemic, dimorphic environmental fungi found in the soil of the southwestern United States, Mexico, and Central and South America (*1*). Clinical infection ranges from asymptomatic to diverse manifestations including pneumonia, soft tissue and osteoarticular infection, meningitis, and disseminated disease (*2*). On the basis of findings from the seminal 1957 seropositivity survey (*3*) that established the commonly accepted geographic distribution of *Coccidioides* in the United States, 6 states were classified as coccidioidomycosis-endemic (Arizona, California, Nevada, New Mexico, Texas, and Utah); California and Arizona had the highest seroprevalence (*4*). On the basis of that study, 3 counties in southwestern Utah were considered coccidioidomycosis-endemic: Iron, Kane, and Washington (*3*). With the exception of reports from a widely publicized 2001 outbreak of coccidioidomycosis at an archeological dig in Uintah County in the US Park Service’s Dinosaur National Monument (*5*–*7*), there are few published data on this disease in Utah. However, recent data suggest that southwestern Utah might represent an area of increased disease burden (*8*). Here we report a description of the epidemiology of coccidioidomycosis in Utah and explore environmental and climatic factors contributing to regional variations in statewide incidence using data from cases reported to the Utah Department of Health (UDOH) during 2009–2015. We also describe clinical characteristics and outcomes using patient-level data from the Intermountain Healthcare System during 2006–2015.

## Methods

### Clinical Characteristics

To describe the clinical characteristics and outcomes of coccidioidomycosis, we used patient-level data from Intermountain Healthcare, an integrated health network with 21 hospitals and 180 clinics in urban and rural Utah. Each year, 1.5 million unique patients, over half of Utah’s 2010 population of 2,763,885 (https://www.census.gov/quickfacts/UT), receive care in the Intermountain Healthcare network. We identified all cases of proven or probable coccidioidomycosis recorded during January 1, 2006–December 31, 2015, by applying a previously published query methodology to clinical data from the Intermountain electronic data warehouse. We used an iterative search process by querying each of 7 different types of clinical and diagnostic data associated with the diagnosis of coccidioidomycosis: codes from the International Classification of Diseases (ICD) 9th (code range 114.x) and 10th (code range B38.x) Revisions, laboratory tests for *Coccidioides*, microbiologic culture data, pathologic data, radiologic data, pharmacy data for antifungal medications, and composite data identifying immunocompromised patients at higher risk for fungal disease (*9*). Laboratory data included serologic assays for *Coccidioides*: IgM/IgG by ELISA, IgM/IgG by immunodiffusion, complement fixation (CF) titers for IgG (ARUP Laboratories, https://www.aruplab.com), and PCR for *Coccidioides* (Mayo Medical Laboratories, https://www.mayocliniclabs.com).

**Figure 1:**
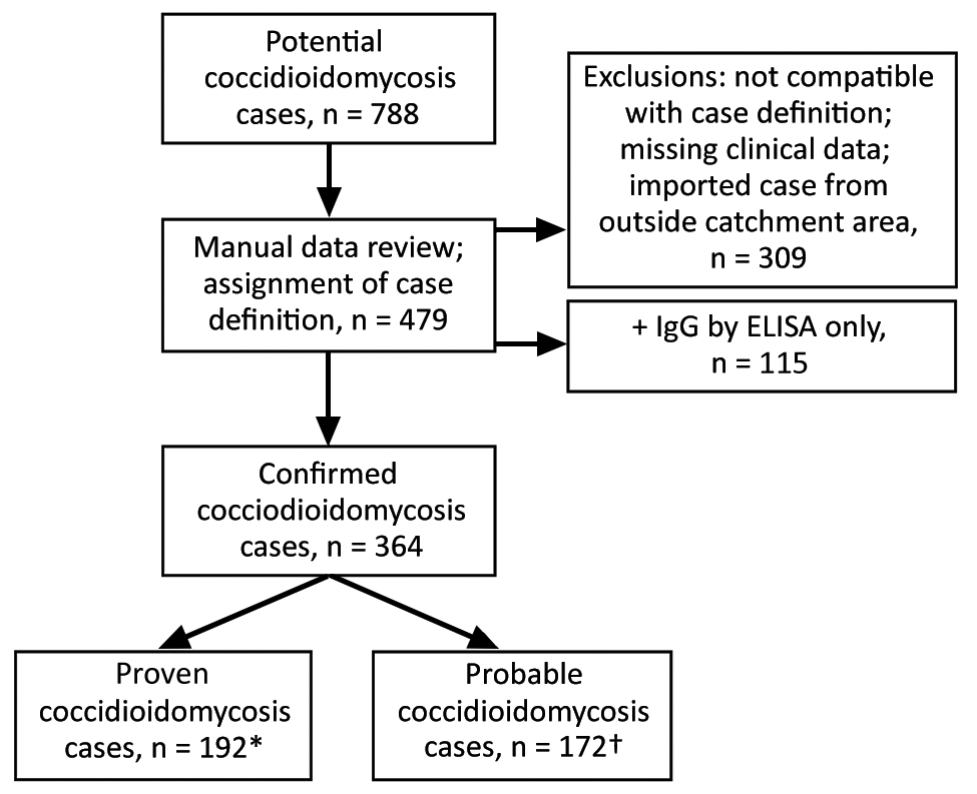
Flowchart showing process for inclusion of possible coccidioidomycosis studies in study of cases in Utah, 2006–2015. Confirmed cases had ≥1 of the following: 1) histopathological, cytopathological, or direct microscopic evidence of *Coccidioides* spherules with tissue damage from sterile specimen or tissue biopsy; 2) culture from any specimen or tissue biopsy positive for *C. immitis* or *C. posadasii*; 3) blood culture positive for *C. immitis* or *C. posadasii;* 4) positive *Coccidioides* serology in cerebrospinal fluid; or 5) two-dilution rise in *Coccidioides* CF titer measured in consecutive blood samples tested concurrently. Probable cases had a *Coccidioides* complement fixation titer >1:2 or positive IgM or IgG by EIA/ELISA or immunodiffusion in the setting of a compatible clinical syndrome and ≥1 of the following: 1) systemic infection with fever, chills, night sweats, weight loss; 2) cutaneous or musculoskeletal infection; 3) pulmonary involvement with nodules, cavitation, hilar lymphadenopathy; 4) meningitis; or 5) visceral infiltration. Definitions based on criteria set by the European Organization for Research and Treatment of Cancer/Invasive Fungal Infections Cooperative Group; National Institute of Allergy and Infectious Diseases Mycoses Study Group (*10*).

We extracted demographic and other clinical data for patients in the Intermountain electronic data warehouse cohort, then manually reviewed all potential cases identified by electronic query to verify the diagnosis by laboratory, microbiologic, and pathologic test results; we validated correlating clinical symptoms using imaging reports, clinical notes, and electronic medical record (EMR) data. We classified each case as proven or probable according to definitions established by the European Organization for Research and Treatment of Cancer/Invasive Fungal Infections Cooperative Group and Mycoses Study Group (EORTC/MSG) (*10*). We considered cases proven if they met ≥1 of the following requirements: histopathologic, cytopathologic, or direct microscopic evidence of *Coccidioides* spherules with tissue damage from sterile specimen or tissue biopsy; culture from any specimen or tissue biopsy positive for *C. immitis* or *C. posadasii*; positive blood culture for *C. immitis* or *C. posadasii*; positive *Coccidioides* serology in cerebrospinal fluid; or 2-dilution rise in *Coccidioides* CF titer measured in consecutive blood samples tested concurrently. We considered cases probable if case-patients had a *Coccidioides* CF titer >1:2 or positive IgM or IgG by enzyme immunoassay (EIA)/ELISA or immunodiffusion in the setting of a compatible clinical syndrome, which could include >1 of the following: 1) systemic infection with fever, chills, night sweats, weight loss; 2) cutaneous or musculoskeletal infection; 3) pulmonary involvement with nodules, cavitation, hilar lymphadenopathy; 4) meningitis; or 5) visceral infiltration. We included case data in the study if the cases met criteria for proven or probable infection (Figure 1).

For the Intermountain Healthcare cohort used for describing clinical characteristics, we included cases from small communities just outside the Utah border for which Intermountain Healthcare facilities serve as the primary access to healthcare. These cases were not included in the cohort used for epidemiologic analyses. We excluded cases in which it was clear from the EMR that the infection was acquired outside of Utah and surrounding communities. We also excluded cases that did not meet the EORTC/MSG definition for proven or probable infection. Because of the higher likelihood of a false positive test with ELISA IgM, we excluded cases if the ELISA IgM was positive but not the ELISA IgG and a diagnosis other than coccidioidomycosis was considered more likely. We also excluded cases with a positive ELISA IgG alone and no corresponding clinical signs or symptoms. We manually confirmed the location of diagnosis and management using the patient’s residential ZIP code from EMRs. If the city of residence was identified but not the ZIP code, we randomly imputed 1 of the ZIP codes corresponding to that city. We reviewed clinical notes for information regarding disease presentation, reasons for testing for coccidioidomycosis, and interpretation of laboratory results by the physician. We also documented whether antifungal drugs were prescribed and the duration of treatment.

### Epidemiologic Analyses

For epidemiologic analyses, we used data from UDOH to ensure we evaluated the entire state population. For this cohort, we included case counts by county by year during 2009–2015. We excluded cases from before 2009 because of acknowledged limitations in data accuracy before that time. As a sensitivity analysis, we compared agreement between results from the case-finding methodology applied to the Intermountain Healthcare data with records from UDOH of cases diagnosed within Intermountain Healthcare facilities.

### Statistical Analysis

Descriptive statistics to compare clinical characteristics were performed using a χ^2^ test for categorical data and the Mann-Whitney U-test for nonnormally distributed continuous data. To compare characteristics between patients with pulmonary and nonpulmonary disease, we developed a logistic regression model including factors significant at an α-significance of <0.1, then reduced it to a parsimonious model. We confirmed the goodness of fit using the Hosmer-Lemeshow method and fitted a simple least-squares linear regression to model the variation in statewide incidence over time.

To explore the association between environmental and anthropological features and geographic variation in observed coccidioidomycosis incidence, we developed a generalized linear model using year, annual population for 2006–2015 (US Census Bureau, https://www.census.gov), PRISM AN81m mean annual air temperature and precipitation (https://prism.oregonstate.edu) (*11*,*12*), and total annual new construction permits per 100,000 population for 2006–2015 (Ivory-Boyer Construction Report and Database, https://gardner.utah.edu/economics/ivory-boyer-construction-database) as covariates. We included year to account for potential fixed-year effects and population to capture differences in signals and levels of cases between urban versus rural counties. We included temperature and precipitation data because both climate factors have been shown to correlate with cases in other endemic regions (*8*,*13*–*16*). Last, we included new construction permits because coccidioidomycosis outbreaks have occurred in areas with construction activity, caused by soil-disrupting activities that increase airborne dust containing *Coccidioides* spp. (*17*–*19*). We also explored the contributions of soil pH (SSURGO database, https://www.nrcs.usda.gov/wps/portal/nrcs/detail/soils/survey/?cid=nrcs142p2_053627) and soil frost-free days and freeze-free intervals (Utah Climate Center, https://climate.usu.edu) but ultimately did not include these in the final model. We assessed model fitness using F-test, R^2^, and residuals. Then, to predict geographic variation in coccidioidomycosis incidence after accounting for environmental and construction factors and the secular trend, we used an analysis of covariance model using county as a fixed variable to estimate the adjusted mean incidence. We input these estimated adjusted incidences into a spatiotemporal geographic information systems model to map predicted incidence by county for the time period. Statistical analysis was conducted using SPSS Statistics 22 (IBM, https://www.ibm.com). This study was approved by the Intermountain Healthcare institutional review board.

## Results

### Demographic and Clinical Data

From the 788 cases we electronically identified initially, 364 patients had serologic, microbiological, or pathological evidence of proven or probable coccidioidomycosis (Figure 1); we excluded an additional 115 patients living in the endemic regions of Utah because they had positive IgG results from ELISA but no evidence of clinical disease. We classified 192 (52.7%) of the 364 cases as proven and 172 (47.3%) as probable (Table 1). Median age of case-patients was 61 years (range 1–97 years); 3.6% were <18 years of age. Over half (55.2%) of patients were male, and 87.9% identified as white. Patients had a median Charlson comorbidity score of 2 (range 0–4); the most common coexisting conditions were chronic pulmonary disease (144, 39.6%), diabetes mellitus (81, 22.3%), and malignancy (76, 20.9%). Only a few patients were taking immunosuppressive medications (27, 7.4%) or undergoing chemotherapy (4, 1.1%) at the time of their diagnosis, and 154 (42.3%) patients were hospitalized for coccidioidomycosis with the length of stay 0–5 days (0 indicating only an emergency room visit); 25.3% of the cohort had ≥1 hospitalization within ≤6 weeks after diagnosis. All-cause mortality was 5.5% at 42 days and 9.1% at 1 year.

**Table 1:** Demographic and clinical data from coccidioidomycosis cases identified in the Intermountain Healthcare system, Utah, USA, 2006–2015*

Variables	No. (%)
Total patients	364
Demographics	
Age, y, median (IQR)	61 (44–72)
Pediatric <18 y	13 (3.6)
Male sex	201 (55.2)
Race	
American Indian	5 (1.4)
Asian	2 (0.5)
Black	1 (0.3)
Hispanic	20 (5.5)
Pacific Islander	9 (2.5)
Unknown	7 (1.9)
White	320 (87.9)
Coexisting conditions/medical factors	
Charlson comorbidity score, median (IQR)	2 (0–4)
Diabetes mellitus	81 (22.3)
Hepatic disease	61 (16.8)
Chronic pulmonary disease	144 (39.6)
Connective tissue disease	27 (7.4)
Congestive heart failure	53 (14.6)
Neurologic disease	52 (14.3)
Renal disease	45 (12.4)
History of malignancy	76 (20.9)
Hematopoietic stem cell transplant	2 (0.5)
Solid organ transplant	2 (0.5)
Any immunosuppressive medication	27 (7.4)
Corticosteroids	25 (6.9)
Anti-TNF	3 (0.8)
Antimetabolite	2 (0.5)
Chemotherapy	4 (1.1)
Laboratory test results	
Lymphopenia, absolute lymphocyte count <500	12 (3.3)
Lymphopenia value at diagnosis, absolute lymphocyte count, median (IQR)	300 (300–400)
Outcomes	
Coccidioidomycosis-related hospital admission	154 (42.3)
Hospital length of stay, d, median (IQR)	0 (0–5)
42-d all-cause mortality	20 (5.5)
1-y all-cause mortality	33 (9.1)
Case classification	
Proven	192 (52.7)
Probable	172 (47.3)

*Values are no. (%) except as indicated. IQR, interquartile range; TNF, tumor necrosis factor.

### Sites of Infection

Primary pulmonary infection was the most common type of infection, in 323 (88.7%) of 364 patients; 4 (1.1%) pulmonary patients had meningitis. Of 41 patients with nonpulmonary disease, 11 (26.8%) had disseminated infection (Table 2; Appendix Table 1). We noted no significant differences in age or coexisting conditions, but did find a trend toward significance (p≤0.05) for chronic neurologic disease, diagnosed in 24.4% (10/41) of nonpulmonary disease patients compared with 13.0% (42/323; p = 0.08) of pulmonary disease patients (Appendix Table 1). Among all those with nonpulmonary disease, 22.2% (9/41) were nonwhite patients, but only 10.8% (35/323; p = 0.05) of patients with pulmonary disease were nonwhite. Nonpulmonary disease was also more common than pulmonary disease in patients receiving immunosuppressive medications, 14.6% (6/41) versus 6.5% (21/323; p = 0.06) and those with lymphopenia preceding diagnosis, 9.8% (4/41) versus 2.5% (8/323; p = 0.04). In a multivariable logistic regression including use of any immunosuppressing medication, neurologic disease, and lymphopenia, only lymphopenia remained a predictor for nonpulmonary disease (OR 4.56, 95% CI 1.2–14.8).

**Table 2:** Clinical features based on case classification from the Intermountain Healthcare system, Utah, USA, 2006–2015

Characteristic	No. (%) cases		
	Total	Proven	Probable
Total	364 (100)	192 (52.7)	172 (47.3)
Primary method of diagnosis			
Laboratory	189 (51.9)	17 (8.9)	172 (100)
Microbiology	43 (11.8)	43 (22.4)	0
Pathology	106 (29.1)	106 (55.2)	0
Microbiology, pathology	26 (7.1)	26 (13.5)	0
Infection site			
Abdomen, peritoneal fluid	1 (0.3)	1 (0.5)	0
Adrenal	2 (0.5)	2 (1.0)	0
Back	1 (0.3)	1 (0.5)	0
Disseminated	11 (3.0)	10 (5.2)	1 (0.6)
Extremity	6 (1.6)	6 (3.1)	0
Liver	1 (0.3)	0	1 (0.6)
Lung only	316 (86.8)	154 (80.2)	162 (94.2)
Lung and lymph node	7 (1.9)	7 (3.6)	0
Lymph node only	4 (1.1)	4 (2.1)	0
Meningitis	4 (1.1)	3 (1.6)	1 (0.6)
Skin	5 (1.4)	3 (1.6)	2 (1.2)
Unknown	6 (1.6)	1 (0.5)	5 (2.9)
Location of diagnosis			
Urgent care/emergency department	12 (3.3)	2 (1.0)	10 (5.8)
Inpatient hospital	110 (30.2)	59 (30.7)	51 (29.7)
Primary care provider	43 (11.8)	22 (11.5)	21 (12.2)
Pulmonary department	84 (23.1)	40 (20.8)	44 (25.6)
Infectious diseases	3 (0.8)	0	3 (1.7)
Surgery department	39 (10.7)	38 (19.8)	1 (0.6)
Other	24 (6.6)	19 (9.9)	5 (2.9)
Unknown	50 (13.7)	12 (6.3)	38 (22.1)
Diagnosed as work-up for malignancy	104 (28.6)	100 (52.1)	4 (2.33)

### Diagnosis and Management

We confirmed a coccidioidomycosis diagnosis with serologic testing in 51.9% of cases and with microbiologic or pathologic evidence of *Coccidioides* in 48.1% of cases (Table 2). Patients were diagnosed in a hospital in 110 (30.2%) cases; among outpatients, 23.1% were diagnosed by a pulmonologist, 11.8% by a primary care provider, 10.7% by a surgeon, and only 0.8% by an infectious disease physician. Of interest, 104 patients (28.6%) were diagnosed as part of a workup for malignancy, usually for an incidental pulmonary nodule. Of the 364 patients in the study, 209 (57.4%) were treated with antifungal therapy alone; 12.6% of case-patients received no surgical or antifungal therapy (Appendix Table 2). Fluconazole (91.7%) was the most common antifungal agent prescribed, followed by amphotericin B (3.2%); 20.8% of patients received >1 different antifungal agent during their treatment.

### Epidemiology and Geographic Variation

**Figure 2:**
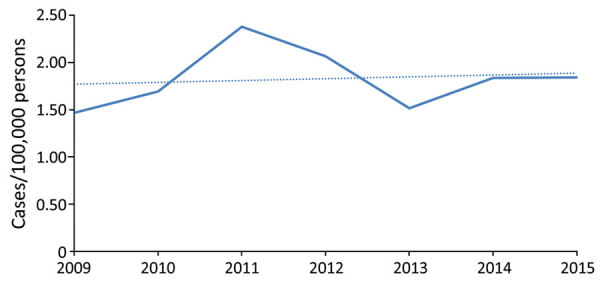
Annual statewide coccidioidomycosis incidence calculated from cases reported to the Utah Department of Health, Utah, 2009–2015. The dotted line represents the line of best fit for the data with an R^2^ of 0.018.

**Figure 3:**
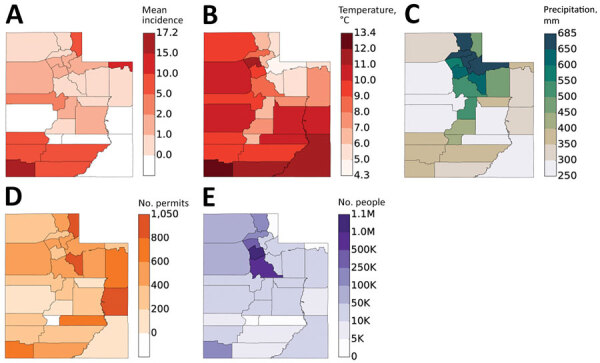
Comparison of mean coccidioidomycosis incidence (per 100,000 population per year) by county, Utah, 2009–2015 (A), with mean annual air temperature (B); precipitation, mm (C); construction permits per 100,000 population (D); and population (E). K, thousand; M, million.

We found 366 cases reported during 2009–2015. Mean observed statewide incidence was 1.83 cases/100,000 population/year; yearly rates increased by a mean of 0.02 cases/100,000 population/year from 2009 through 2015 (R^2^ = 0.018, Figure 2). Washington County, in the southwestern part of the state, accounted for the largest proportion (47.5%) of cases, a mean observed incidence of 17.2 cases/100,000 population/year (Figure 3, Table 3). Outside of Washington County, incidence was next highest in the adjacent southwestern counties of Beaver, Garfield, Iron, and Kane, then in Daggett and Rich Counties in the northeast corner of the state (Table 3; Figure 3). In the generalized linear model accounting for temporal trend, the factors that best explained regional variation in observed incidence included population (effect size [partial η^2^] 0.068, p = 0.001), mean air temperature (effect size 0.246; p<0.001), and new construction permits/100,000 population (effect size 0.072; p = 0.001), but precipitation was not significantly associated (effect 0.022; p = 0.059; R^2^ = 0.42) (Appendix Table 3).

**Table 3:** Distribution by county of coccidioidomycosis cases reported to the Utah Department of Health, Utah, USA, 2009–2015

County*	Total (%)†	Observed mean cases/year	Mean incidence/100,000 population/year	
			Observed	Estimated
Washington	174 (47.5)	24.9	17.2	17.2
Salt Lake	79 (21.6)	11.3	1.06	–0.01
Davis	23 (6.3)	3.29	1.02	–0.11
Utah	21 (5.7)	3.00	0.56	1.00
Iron	17 (4.6)	2.43	5.23	4.18
Weber	10 (2.7)	1.43	0.60	–0.79
Tooele	6 (1.6)	0.86	1.43	0.65
Summit	5 (1.4)	0.71	1.93	4.57
Beaver	4 (1.1)	0.57	8.83	6.93
Cache	4 (1.1)	0.57	0.50	1.14
Garfield	3 (0.8)	0.43	8.44	7.32
Box Elder	2 (0.5)	0.29	0.85	–0.03
Kane	2 (0.5)	0.29	5.95	4.73
Juab	2 (0.5)	0.29	2.78	0.64
Sanpete	2 (0.5)	0.29	1.01	0.20
Uintah	2 (0.5)	0.29	0.80	2.70
Carbon	1 (0.3)	0.14	0.68	–0.88
Daggett	1 (0.3)	0.14	12.9	13.7
Duchesne	1 (0.3)	0.14	0.71	2.62
Emery	1 (0.3)	0.14	1.34	–1.00
Morgan	1 (0.3)	0.14	1.40	2.43
Rich	1 (0.3)	0.14	6.33	11.0
Sevier	1 (0.3)	0.14	0.69	–0.98
Wasatch	1 (0.3)	0.14	0.58	5.66
Utah	366‡ (100)	52.3	1.83	3.45

*Counties with no cases included in the table: Grand, Millard, Piute, San Juan, and Wayne. †Two cases in 2015 could not be classified by county. ‡Data from analysis of covariance model adjusting for year, mean annual temperature, mean annual precipitation, mean annual population, and mean number of new construction permits/100,000 population/year.

For the analysis of covariance model, in which we used county as a fixed effect and adjusted by secular trend, population, mean annual temperature, precipitation, and new construction permits, the estimated mean statewide incidence was 3.45 cases/100,000 population/year (R^2^ = 0.92) (Table 3; Appendix Table 4). In this model, estimated adjusted mean incidence was highest in Washington County at 17.2 cases/100,000 population per year. The estimated incidence was higher than the observed incidence in Summit, Uintah, Duchesne, Morgan, and Rich Counties in northeastern Utah (Table 3).

## Discussion

These data, representing the results of a modern epidemiologic study, confirm coccidioidomycosis as a clinically relevant endemic mycosis in Utah. Our analyses benefited from the granularity of patient-level data combined with UDOH statewide data. Although not on the scale of incidence reported for Arizona (154.6 cases/100,000 population/year) or California (9.37 cases/100,000 population/year) (*20*–*22*), the incidence (1.83 cases/100,000 population/year) in Utah during 2009–2015 was higher than previously reported, and Utah ranks as the third most endemic state (*4*).

Coccidioidomycosis clusters regionally within the state. Washington, Garfield, Beaver, Kane, and Iron Counties in the southwestern portion of the state account for the most cases. Although regional climate contributes to this distribution, rapid population growth and new construction in this area of the state might also play a role. As of 2018, St. George, located in Washington County, was one of the fastest growing metropolitan areas in the United States (US Census Bureau). Residential and commercial construction disrupts soil and exposes residents to aerosolized arthroconidia, increasing the risk for contracting coccidioidomycosis (*19*,*23*). With increasing population growth in this area, we hypothesize that the rate of coccidioidomycosis incidence will also continue to rise. Future studies focusing on incidence among construction workers or residents living in areas with increased rates of construction will be key to further understand this association. Washington County also represents a large recreational area for travelers, both those commuting to other destinations in the Interstate 15 corridor and those traveling to Zion National Park, the fourth-most visited national park in the United States in 2018, which averages 4.3 million visitors/year (National Park Services Visitor Use Statistics, https://irma.nps.gov/STATS).

Of additional interest, when climate, population, and construction factors were taken into account, our model predicted a second hotspot for future high coccidioidomycosis incidence in the northeastern corner of the state. Although the current observed incidence in these counties is low, they are also sparsely populated but with substantial population growth expected, the incidence in these areas might also be expected to increase. This finding was especially intriguing in the context of the 2001 coccidioidomycosis outbreak (adjusted mean incidence: 2.70 cases/100,000 population/year) that occurred in Uintah County in northeast Utah, which includes part of Dinosaur National Monument.

In addition to the factors that we included in our analyses, other environmental factors such as soil pH and composition and the geographic distribution of small mammal species important in the lifecycle of *Coccidioides* (*24*) might also contribute to the geographic variation in disease incidence and merit additional research. Future studies including PCR testing of soil and air samples will be important to clarify the interactions between the environment and *Coccidioides* pathogens and enable more accurate epidemiologic forecasting.

The observed all-cause mortality in the study cohort was higher than reported in an earlier study (*25*). Because roughly one third of cases in our study were diagnosed in a hospital, delay in diagnosis because of lack of clinical awareness might have led to increased death. In addition, because of this finding of elevated death rates, potential differences in virulence among *Coccidioides* strains circulating in Utah should be considered to better understand this phenomenon (*26*). Congruent with findings from prior studies (*27*), persons of non-White race and those taking immunosuppressive medications were more likely to have the nonpulmonary form of the disease.

Given the high incidence in southwestern Utah, more widespread efforts to educate clinicians about coccidioidomycosis are urgently needed, especially as the population increases and ages. In these areas, where pulmonary and infectious diseases specialists are scarce, primary care providers and those working in urgent care settings serve as the front line for diagnosing and treating diseases such as coccidioidomycosis. Because nearly one third of patients in this cohort were diagnosed as part of a workup for malignancy, our findings suggest that additional awareness efforts should be targeted to the hematologists and oncologists serving a broad referral area in Utah, Nevada, and Arizona. Radiologists should also be included so that they might consider coccidioidomycosis as a differential diagnosis in the presence of relevant radiological features.

An additional 115 patients living in southwestern Utah were excluded from the study although they had positive IgG ELISA results because of a lack of clinical disease evidence. It is unclear if these cases represent temporally remote or subclinical exposures with long-lasting seropositivity, questioning the current paradigm that *Coccidioides* IgG wanes over time. Additional analysis of that subgroup of the cohort will need to be conducted to further understand this phenomenon.

Our study’s first limitation is that the reported demographic and clinical data are based on the subset of cases from Intermountain Healthcare identified within the state, but incidence data are based on cases reported to the state health department. When we manually compared Intermountain Healthcare patient-level data with statewide reportable disease data from UDOH for the same cases, there were differences, particularly for case confirmation and regional distribution (e.g., more reportable cases in northern counties). This might have been because of decreased specificity related to the granularity of laboratory-initiated health department data and decreased sensitivity in Intermountain Healthcare data, where not all possible cases might have been detected. For example, not all physicians used EMR, and in some cases clinical data were missing; therefore, we excluded those cases to maintain data integrity. This process likely led to an underestimation of the true number of cases within the state. Third, we used ZIP code information as a surrogate for the location of disease acquisition. Without a direct survey of patients to elucidate occupational and recreational exposures, this might skew the distribution of disease across the state. Last, we excluded cases from the demographic and clinical analyses when *Coccidioides* CF was positive at 1:2 titer, although UDOH includes cases with CF positive at that titer. However, manual review revealed only 2 cases excluded with an exact 1:2 titer without another positive serologic result. One case had missing clinical information that did not permit us to confirm symptomatology, and 1 case was imported from outside of the state.

In conclusion, we found that coccidioidomycosis incidence in Utah is higher than previously described and clusters primarily in the recognized endemic area in the southwestern part of the state. However, in geospatial modeling accounting for environmental factors, we identified a second potential area in the northeast that might have conditions conducive to future increases in *Coccidioides* incidence. Increasing the awareness of front-line providers and especially oncologists in southwestern Utah is necessary for early recognition and clinical management of the disease, but enhanced clinical surveillance in the northeast might increase case detection. Serologic and environmental testing might further elucidate distribution of *Coccidioides* organisms and determine the effects of air temperature, population growth, and construction on coccidioidomycosis disease burden in the state.

Appendix Additional information on epidemiology, clinical features, and outcomes of coccidioidomycosis, Utah, 2006–2015.
