# Supplementary material for: Epidemiology, Clinical Features, and Outcomes of Coccidioidomycosis, Utah, 2006–2015
Source: Emerg Infect Dis. 2021 Sep;27(9):2269–77. doi: 10.3201/eid2709.210751 (PMC8386810; doi:10.3201/eid2709.210751)
Supplement: Appendix — Additional information on epidemiology, clinical features, and outcomes of coccidioidomycosis, Utah, 2006–2015. [file 21-0751-Techapp-s1.pdf]

Article DOI: https://doi.org/10.3201/eid2709.210751

# Epidemiology, Clinical Features, and Outcomes of Coccidioidomycosis, Utah, 2006–2015

## Appendix

**Appendix Table 1.** Demographic and clinical data of pulmonary vs. nonpulmonary disease patients from coccidioidomycosis cases identified in the Intermountain Healthcare System database, 2006–2015 (N = 366)*

| Characteristic | Pulmonary disease n (%) | Nonpulmonary disease n (%) | P value |
|---|---|---|---|
| Total cases | 323 (88.7) | 41 (11.3) | NA |
| Age | 61 (44–71)† | 60 (40–74)† | 0.94 |
| Pediatric | 12 (3.7) | 1 (2.4) | 1.00 |
| Male | 178 (55.1) | 23 (56.1) | 1.00 |
| Race | | | |
| American Indian | 3 (0.9) | 2 (4.9) | 0.014 |
| Asian | 2 (0.6) | 0 (0.0) | |
| Black | 0 | 1 (2.4) | |
| Hispanic | 18 (5.6) | 2 (4.9) | |
| Pacific Islander | 7 (2.2) | 2 (4.9) | |
| Unknown | 5 (1.5) | 2 (4.9) | |
| White | 288 (89.2) | 32 (78.0) | |
| All nonwhite | 35 (10.8) | 9 (22.0) | 0.046 |
| White | 288 (89.2) | 32 (78.0) | |
| Charlson comorbidity score | 2 (0–3)† | 2 (0–5)† | 0.32 |
| Additional medical factors | | | |
| Diabetes mellitus | 70 (21.7) | 11 (26.8) | 0.58 |
| Hepatic disease | 53 (16.4) | 8 (19.5) | 0.78 |
| Chronic pulmonary disease | 130 (40.2) | 14 (34.1) | 0.56 |
| Connective tissue disease | 22 (6.8) | 5 (12.2) | 0.34 |
| Congestive heart failure | 45 (13.9) | 8 (19.5) | 0.47 |
| Neurological disease | 42 (13.0) | 10 (24.4) | 0.08 |
| Renal disease | 37 (11.5) | 8 (19.5) | 0.22 |
| History of malignancy | 68 (21.1) | 8 (19.5) | 1.00 |
| Hematopoietic stem cell transplant | 2 (0.6) | 0 (0.0) | 1.00 |
| Solid organ transplant | 1 (0.3) | 1 (2.4) | 0.21 |
| Medication | | | |
| Any immunosuppressive medication | 21 (6.5) | 6 (14.6) | 0.06 |
| Corticosteroids | 20 (6.2) | 5 (12.2) | 0.18 |
| Tumor necrosis factor inhibitors | 2 (0.6) | 1 (2.4) | 0.3 |
| Antimetabolite | 1 (0.3) | 1 (2.4) | 0.22 |
| Chemotherapy | 3 (0.9) | 1 (2.4) | 0.38 |
| Lymphopenia, absolute lymphocyte count <500 | 8 (2.5) | 4 (9.8) | 0.04 |
| Outcomes | | | |
| Coccidioidomycosis-related hospital admission | 131 (40.6) | 23 (56.1) | 0.08 |
| Hospital length of stay | 0 (0–4)† | 3 (0–10)† | 0.023 |
| 42-day all-cause mortality | 17 (5.3) | 3 (7.3) | 0.71 |
| 1-year all-cause mortality | 26 (8.0) | 7 (17.1) | 0.08 |
| Case classification | | | |
| Proven | 161 (49.8) | 31 (75.6) | 0.003 |
| Probable | 162 (50.2) | 10 (24.4) | 0.003 |

*NA, not applicable
†Median (25%–75% interquartile range), not number (%)

**Appendix Table 2.** Coccidioidomycosis management of coccidioidomycosis case-patients identified in the Intermountain Healthcare System database, 2006–2015 (N = 366)

| Management | n (%) |
|---|---|
| Treatment options used | |
| Antifungal alone | 209 (57.4) |
| Antifungal + surgical resection | 7 (1.9) |
| Surgical resection alone | 17 (4.7) |
| No treatment | 46 (12.6) |
| Unknown | 85 (23.4) |
| Initial antifungal used | 216 |
| Amphotericin B | 7 (3.2) |
| Caspofungin | 2 (0.9) |
| Fluconazole | 198 (91.7) |
| Itraconazole | 2 (0.9) |
| Micafungin | 4 (1.9) |
| Posaconazole | 1 (0.5) |
| Voriconazole | 1 (0.5) |
| Unknown | 1 (0.5) |
| Switched to a different regimen | 45 (20.8) |
| Duration of treatment | |
| Unknown duration | 61 (28.2) |
| <1 mo | 17 (7.9) |
| 1 to <3 mo | 5 (2.3) |
| 3 to <6 mo | 23 (10.6) |
| 6 to <9 mon | 35 (16.2) |
| 9 to <12 mo | 19 (8.8) |
| ≥12 mo | 44 (20.4) |
| Lifetime treatment | 12 (5.6) |
| Managing physician | |
| Primary care provider | 30 (8.2) |
| Pulmonary specialist | 122 (33.5) |
| Infectious disease specialist | 83 (22.8) |
| Other | 24 (6.6) |
| Unknown | 107 (29.4) |

**Appendix Table 3.** Generalized linear model for analysis of variation of coccidioidomycosis cases reported to the Utah Department of Health by year, population, temperature, precipitation, and construction*

| Variable | Type III sum of squares | DF | Mean square | F value | P value | Partial $\eta^2$ |
|---|---|---|---|---|---|---|
| Corrected model | 2,094.654† | 5 | 418.931 | 22.913 | 0.000 | 0.414 |
| Intercept | 19.040 | 1 | 19.040 | 1.041 | 0.309 | 0.006 |
| Year | 20.508 | 1 | 20.508 | 1.122 | 0.291 | 0.007 |
| Mean annual population | 214.951 | 1 | 214.951 | 11.756 | 0.001 | 0.068 |
| Mean annual temperature | 966.697 | 1 | 966.697 | 52.872 | <0.001 | 0.246 |
| Mean annual precipitation | 66.264 | 1 | 66.264 | 3.624 | 0.059 | 0.022 |
| Mean annual new construction permits/100,000 | 229.484 | 1 | 229.484 | 12.551 | 0.001 | 0.072 |
| Error | 2,961.989 | 162 | 18.284 | NA | NA | NA |
| Total | 5,854.000 | 168 | NA | NA | NA | NA |
| Corrected total | 5,056.643 | 167 | NA | NA | NA | NA |

*DF, degrees of freedom; NA, not applicable
†$R^2$ = 0.42 (adjusted $R^2$ = 0.396).

**Appendix Table 4.** ANCOVA for estimated adjusted coccidioidomycosis incidence calculated from cases reported to the Utah Department of Health (N = 5,854) with covariates of year, population, temperature, precipitation, construction and county*

| Covariate | Type III sum of squares | DF | Mean square | F value | P value | Partial $\eta^2$ |
|---|---|---|---|---|---|---|
| Corrected model | 4,644.421 | 28 | 165.872 | 55.932 | 0.000 | 0.918 |
| Intercept | 0.576 | 1 | 0.576 | 0.194 | 0.660 | 0.001 |
| Year | 0.604 | 1 | 0.604 | 0.204 | 0.652 | 0.001 |
| Mean annual population | 7.485 | 1 | 7.485 | 2.524 | 0.479 | 0.018 |
| Mean annual temperature | 1.492 | 1 | 1.492 | 0.503 | 0.903 | 0.004 |
| Mean annual precipitation | 0.044 | 1 | 0.044 | 0.015 | 0.518 | 0.000 |
| Mean annual new construction permits/100,000 | 1.248 | 1 | 1.248 | 0.421 | 0.114 | 0.003 |
| County | 2,549.767 | 23 | 110.859 | 37.381 | 0.000 | 0.861 |
| Error | 412.222 | 139 | 2.966 | NA | NA | NA |
| Total | 5,854.000 | 168 | NA | NA | NA | NA |
| Corrected total | 5,056.643 | 167 | NA | NA | NA | NA |

*DF, degrees of freedom; NA, not applicable

**Appendix Table 5.** Distribution of coccidioidomycosis cases identified in the Intermountain Healthcare System (N = 366) by zip code, and Utah city and county

| Zip Code | n (%) | City | County |
|---|---|---|---|
| 84790 | 60 (16.48) | St. George | Washington |
| 84770 | 53 (14.56) | St. George | Washington |
| 84780 | 38 (10.44) | Washington | Washington |
| 89027 | 25 (6.87) | Mesquite | Clark |
| 84737 | 22 (6.04) | Hurricane | Washington |
| 89024 | 10 2.75) | Mesquite | Clark |
| 84765 | 8 (2.20) | Santa Clara | Washington |
| 84721 | 8 (2.20) | Cedar City | Iron |
| 84738 | 6 (1.65) | Ivins | Washington |
| 84720 | 6 (1.65) | Cedar City | Iron |
| 86432 | 5 (1.37) | Little Field | Mohave |
| 84121 | 5 (1.37) | Salt Lake City | Salt Lake |
| 89034 | 4 (1.10) | Mesquite | Clark |
| 89040 | 3 (0.82) | Overton | Clark |
| 86021 | 3 (0.82) | Colorado City | Mohave |
| 84020 | 3 (0.82) | Draper | Salt Lake |
| 84092 | 3 (0.82) | Sandy | Salt Lake |
| 84003 | 3 (0.82) | American Fork | Utah |
| 84651 | 3 (0.82) | Payson | Utah |
| 84745 | 2 (0.55) | La Verkin | Washington |
| 84771 | 2 (0.55) | St. George | Washington |
| 84784 | 2 (0.55) | Hildale | Washington |
| 84713 | 2 (0.55) | Beaver | Beaver |
| 84501 | 2 (0.55) | Price | Carbon |
| 86022 | 2 (0.55) | Fredonia | Coconino |
| 84014 | 2 (0.55) | Centerville | Davis |
| 84041 | 2 (0.55) | Layton | Davis |
| 84761 | 2 (0.55) | Parowan | Iron |
| 84047 | 2 (0.55) | Midvale | Salt Lake |
| 84070 | 2 (0.55) | Sandy | Salt Lake |
| 84093 | 2 (0.55) | Sandy | Salt Lake |
| 84109 | 2 (0.55) | Salt Lake City | Salt Lake |
| 84116 | 2 (0.55) | Salt Lake City | Salt Lake |
| 84118 | 2 (0.55) | Salt Lake City | Salt Lake |
| 84005 | 2 (0.55) | Eagle Mountain | Utah |
| 84042 | 2 (0.55) | Lindon | Utah |
| 84043 | 2 (0.55) | Lehi | Utah |
| 84097 | 2 (0.55) | Orem | Utah |
| 84604 | 2 (0.55) | Provo | Utah |
| 84032 | 2 (0.55) | Heber | Wasatch |
| 84401 | 2 (0.55) | Ogden | Weber |
| 84405 | 2 (0.55) | Ogden | Weber |
| 84722 | 1 (0.27) | Central | Washington |
| 84757 | 1 (0.27) | New Harmony | Washington |
| 84774 | 1 (0.27) | Toquerville | Washington |
| 84779 | 1 (0.27) | Virgin | Washington |
| 84783 | 1 (0.27) | Dammeron Valley | Washington |
| 84752 | 1 (0.27) | Minersville | Beaver |
| 84302 | 1 (0.27) | Brigham City | Box Elder |
| 89021 | 1 (0.27) | Logandale | Clark |
| 89042 | 1 (0.27) | Panaca | Clark |
| 86040 | 1 (0.27) | Page | Coconino |
| 84010 | 1 (0.27) | Woods Cross/Bountiful | Davis |
| 84015 | 1 (0.27) | Clearfield | Davis |
| 84025 | 1 (0.27) | Farmington | Davis |
| 84037 | 1 (0.27) | Kaysville | Davis |
| 84075 | 1 (0.27) | Syracuse | Davis |
| 84089 | 1 (0.27) | Clearfield | Davis |
| 84726 | 1 (0.27) | Escalante | Garfield |
| 84759 | 1 (0.27) | Panguitch | Garfield |
| 84764 | 1 (0.27) | Bryce | Garfield |
| 84756 | 1 (0.27) | Newcastle | Iron |
| 86406 | 1 (0.27) | Lake Havasu City | Mohave |
| 84044 | 1 (0.27) | Magna | Salt Lake |
| 84054 | 1 (0.27) | North Salt Lake | Salt Lake |
| 84094 | 1 (0.27) | Sandy | Salt Lake |
| 84095 | 1 (0.27) | South Jordan | Salt Lake |

| Zip Code | n (%) | City | County |
|---|---|---|---|
| 84096 | 1 (0.27) | Herriman | Salt Lake |
| 84106 | 1 (0.27) | Salt Lake City | Salt Lake |
| 84108 | 1 (0.27) | Salt Lake City | Salt Lake |
| 84117 | 1 (0.27) | Salt Lake City | Salt Lake |
| 84119 | 1 (0.27) | Salt Lake City | Salt Lake |
| 84120 | 1 (0.27) | Salt Lake City | Salt Lake |
| 84128 | 1 (0.27) | Salt Lake City | Salt Lake |
| 84129 | 1 (0.27) | Salt Lake City | Salt Lake |
| 84165 | 1 (0.27) | Salt Lake City | Salt Lake |
| 84629 | 1 (0.27) | Fairview | Sanpete |
| 84643 | 1 (0.27) | Mayfield | Sanpete |
| 84647 | 1 (0.27) | Mount Pleasant | Sanpete |
| 84754 | 1 (0.27) | Monroe | Sevier |
| 84060 | 1 (0.27) | Park City | Summit |
| 84074 | 1 (0.27) | Tooele | Tooele |
| 84004 | 1 (0.27) | Alpine | Utah |
| 84057 | 1 (0.27) | Orem | Utah |
| 84062 | 1 (0.27) | Pleasant Grove | Utah |
| 84601 | 1 (0.27) | Provo | Utah |
| 84653 | 1 (0.27) | Salem | Utah |
| 84655 | 1 (0.27) | Santaquin | Utah |
| 84715 | 1 (0.27) | Bicknell | Wayne |
| 84067 | 1 (0.27) | Roy | Weber |
| 84317 | 1 (0.27) | Huntsville | Weber |
| 84404 | 1 (0.27) | Ogden | Weber |
